# Supplementary material for: Chemical reactivity theory to analyze possible toxicity of microplastics: Polyethylene and polyester as examples
Source: PLoS One. 2024 Mar 6;19(3):e0285515. doi: 10.1371/journal.pone.0285515 (PMC10917325; doi:10.1371/journal.pone.0285515)
Supplement: Attachment — Submitted filename: letterPLOSONEmartinezRESPONSE.docx [file pone.0285515.s001.docx]

PONE-D-23-12236

Old title: *Microplastics found in Antarctic penguins: chemical reactivity theory to analyze possible toxicity.*

New Title: *Chemical reactivity theory to analyze possible toxicity of microplastics: polyethylene and polyester as examples.*

Dear Arumugam Sundaramanickam, PhD

Academic Editor

PLOS ONE

Please find enclose the revised version of the manuscript entitled *Chemical reactivity theory to analyze possible toxicity of microplastics: polyethylene and polyester as examples.* In what follows we respond to each point raised by the academic editor and reviewers. All questions were answered to the best of our ability.

We hope you find this version suitable for publication.

Kind regards,

Prof. Ana Martínez

UNAM

**Reviewer #1:**

In the abstract, before stating the percentage of polymers, it is better to explain the sources of pollution in the region. Penguins were mentioned in the title, but no results were given regarding the number of microplastics and their size in the study area. How were polymers identified?

**Author's response:**

Following Reviewer #2's suggestion, we removed all information regarding penguins. Therefore, this information is not necessary.

**Reviewer #1:**

Fig 1- Which one is polyethylene and which one PET? Show in lowercase letters.

**Author's response:**

Figure 1 was modified accordingly.

**Reviewer #1:**

The discussion part of the results less compared with other studies.

**Author's response:**

We modified the discussion accordingly

**Reviewer #1:**

The conclusion is very similar to the abstract.

**Author's response:**

We modified the abstract as follows:

Micro- and nanoplastics are widespread throughout the world. In particular, polyethylene (PE) and polyethylene terephthalate or polyester (PET) are two of the most common polymers, used as plastic bags and textiles. To analyze the toxicity of these two polymers, oligomers with different numbers of units were used as models. The use of oligomers as polymeric templates has been used previously with success. We started with the monomer and continued with different oligomers until the chain length was greater than two nm. According to the results of quantum chemistry, PET is a better oxidant than PE, since it is a better electron acceptor. Additionally, PET has negatively charged oxygen atoms and can promote stronger interactions than PE with other molecules. We found that PET forms stable complexes and can dissociate the guanine-cytosine nucleobase pair. This could affect DNA replication. These preliminary theoretical results may help elucidate the potential harm of micro- and nanoplastics.

**Reviewer #2:**

However, the authors focus on only two polymers found in penguin species (based on a single study) but they do not discuss why it is important in the context. The chemical reactivity theory can be applied to any context (I.e., any plastic polymer) and the results here can be associated to any organisms). What is the link to penguins rather than other species in which PE and PET have been found? If penguins are key in this study, the authors should first review all current literature on microplastic contamination in penguins and enlarge their study to depict a more comprehensive toxicity assessment. Moreover, the authors discuss specific effects on DNA, which again can be referred to any biological system. What are the implications for penguin populations and Antarctic ecosystems? If the findings are too general, I suggest to simply remove penguins from the title.

**Author's response:**

We agree with this statement. Penguins are not key to this research. We included them because one of us found microplastics in penguins, but this is no reason to preserve the idea of penguins. In this new version we eliminate all information related to penguins.

**Reviewer #2:**

The article readability can be improved by rechecking the grammar and trying to build more articulated sentences, which sometimes are very short. Some errors are reported below: (not having line numbers made the revision more difficult)

**Author's response:**

We corrected the errors and review the grammar from the beginning.

**Reviewer #2:**

This study is based on citation n.27 (3 penguins species), but there are several studies on the subject, which should be at least mentioned on the introduction, then the authors should explain why they chose to focus only on findings from citation 27 (based on more samples? from more sampling sites?), as it is likely that penguins are not exposed to PE and PET only:

https://doi.org/10.1038/s41598-019-50621-2
https://doi.org/10.1038/s41598-023-39844-6
https://doi.org/10.1016/j.envint.2019.105303
No microplastic were also found in emperor penguins, this should be mentioned in the introduction as well: <https://doi.org/10.1016/j.scitotenv.2022.158314>

**Author's response:**

Following the suggestion of this reviewer, we delete all information about pinguins.

**Reviewer #2:**

“There is also no theoretical research on potential toxicity of these polymers” There are model studies available on in vitro nanoplastic—cell interactions (e.g., <https://doi.org/10.1021/jz402234c>), although based on a different approach. I suggest listing some examples in which chemical reactivity theory has been applied to environmental risk assessment.

**Author's response:**

Following this suggestion, we added more references and the following paragraph:

There are some publications on molecular simulations to determine the effect of nanometric polystyrene particles [32] and the theory of chemical reactivity has been used to study the environmental risk [33-35] of different substances, but there are no studies on oxidative stress or the direct interactions of nanoplastics with DNA nitrogen bases. For this reason, …

New references:

32. Rossi G, Barnoud J, Monticelli L. Polystyrene Nanoparticles Perturb Lipid Membranes

J. Phys. Chem. Lett. 2014; 5, 241−246. dx.doi.org/10.1021/jz402234c

33. Wang Q, Zhang Y, Rogers WJ, Mannan MS. Molecular simulation studies on chemical reactivity of methylcyclopentadiene. Journal of Hazardous Materials 2009; 165, 141–147 doi: 10.1016/j.jhazmat.2008.09.087

34. Rawat P, Singh RN. Experimental and theoretical study of 4-formyl pyrrole derived aroylhydrazones. Journal of Molecular Structure 2015; 1084, 326–339 http://dx.doi.org/10.1016/j.molstruc.2014.12.045

35. Villaverde JJ López-Goti C, Alcamí M, Lamsabhi AM, Alonso-Pradosa JL, Sandín-España P. Quantum chemistry in environmental pesticide risk assessment. Pest. Manag. Sci. 2017; 73: 2199–2202 doi: 10.1002/ps.4641

**Reviewer #2:**

“The results of this research help…” change to: can help
“…and may determine which of the two plastics found in penguins is potentially more dangerous.” Please specify that you are referring to two types plastic polymers based one study.

…until the chain length is greater than 2 nm” change to: was
Rephrase to: In Figures 3 and 4 the optimized structures of the models investigated are shown. The correspondent length of the oligomer is also reported.

“As was explained in the introduction, apparently micro- and nanoplastics may induce oxidative stress. To investigate possible oxidative stress, …” Rephrase to, deleting previous phrase: To investigate possible oxidative stress caused by micro- and nanoplastics,…

In page 6 there are some small errors to correct, please check the text again. Please avoiding the use of English possessive (‘s) with polymers.

“The idea is to…” I suggest to change, continuing the previous sentence with as: in order to. “Polyester can oxidize nucleobases and therefore, microplastics of PET can be harmful.

Change “obvious” to: clear
Change “say” to: state
“no toxic effects of micro and nano- have been observed”. I suggest to add: considering the diversity of biological systems.
“…for three reasons”. I suggest to list the reasons from the least to the most harmful, as in the abstract.
“Although it was found that penguins have…” change “have to: can ingest

**Author's response:**

We made all these correction in this new version. We delete all information concerning penguins.

**Reviewer #2:**

“… we calculated adenine, thymine, cytosine and guanine.” In what sense? Add details

**Author's response:**

To answer this question, we modified the paragraph as follows:

we optimized geometries of adenine, thymine, cytosine and guanin, and we calculated the electron transfer properties.

**Reviewer #2:**

Although it was found that penguins have less polyester than polyethylene, nucleobases might transfer electrons to polyester and therefore it could affect more pinguin’s health.” Add details referred to this statement with actual examples of previous studies showing significant damages following exposure to PET microplastics to support your findings.

**Author's response:**

We delete all information about pinguins so this correction is not necessary.

**Reviewer #2:**

I suggest to add reference/s to page 9 to support the statements. The same applies to page 11: there are many in vitro/in vivo studies with model micro and nanoplastics, the authors should refer to those for comparison and to support their hypothesis.

**Author's response:**

We performed a review of the literature and found no evidence on the comparison of toxicity of PP and PET. I'm afraid I don't understand what evidence the reviewer is suggesting, but I'm interested in following this suggestion as soon as I get the idea.

**Reviewer #2:**

“Micro- and nanoplastics can have long-term stability in various biological media (please add examples of biological media)

**Author's response:**

This is a mistake. We were thinking on the environment. The new sentence is as follows:

“Micro- and nanoplastics can have long-term stability under environmental conditions.

**Reviewer #2:**

As in this study only PE and PET are considered and no mention of the biological system, I suggest the authors to refer to their results are first or preliminary.

**Author's response:**

We added this sentence at the end of the abstract and conclusions.
