# Supplementary material for: Chemical reactivity theory to analyze possible toxicity of microplastics: Polyethylene and polyester as examples
Source: PLoS One. 2024 Mar 6;19(3):e0285515. doi: 10.1371/journal.pone.0285515 (PMC10917325; doi:10.1371/journal.pone.0285515)
Supplement: Attachment — Submitted filename: letterPLOSONEmartinezRESPONSE2.docx [file pone.0285515.s002.docx]

PONE-D-23-12236R2

*Chemical reactivity theory to analyze possible toxicity of microplastics: polyethylene and polyester as examples.*

Dear Arumugam Sundaramanickam, PhD

Academic Editor

PLOS ONE

Please find enclose the revised version of the manuscript entitled *Chemical reactivity theory to analyze possible toxicity of microplastics: polyethylene and polyester as examples.* In what follows we respond to each point raised by the academic editor and reviewers. All questions were answered to the best of our ability.

We hope you find this version suitable for publication.

Kind regards,

Prof. Ana Martínez

UNAM

**Reviewer #1:**

Authors have responded effectively to the initial querries raised. Since the necessary corrections have been made, it is appropriate to publish it as it is.

**Reviewer #2:**

"...there are no studies on oxidative stress or the direct interactions of nanoplastics with DNA nitrogen bases"

this should be changed as there are several studies on ox stress and nanoplastics, for example: <https://doi.org/10.1016/j.envint.2023.107899>; <https://doi.org/10.3389/fpls.2022.1027608>

<https://doi.org/10.3390/antiox11020193>, and as reviewed in <https://doi.org/10.1016/j.scitotenv.2023.163144>

**Author's response:**

They are very interesting references, which are now included in this new version. The following paragraph is also added.

There are also studies on oxidative stress and nanoplastics [36-40] but there are not theoretical studies on oxidative stress or the direct interactions of nanoplastics with DNA nitrogen bases.

36. Ekner-Grzyb A, Duka A, Grzyb T, Lopes I, Chmielowska-Ba ̨k J. Plants oxidative response to nanoplastic. Front. Plant Sci. 2022, 13:1027608. doi: 10.3389/fpls.2022.1027608

37. Ferrante MC, Monnolo A, Del Piano F, Mattace Raso G, Meli R. The pressing issue of micro- and nanoplastic contamination: profiling the reproductive alterations mediated by oxidative stress. Antioxidants 2022, 11: 193 https://doi.org/10.3390/ antiox11020193

38. Ding R, Ma Y, Li T, Sun M, Sun Z, Duan J. The detrimental effects of micro-and nano-plastics on digestive system: An overview of oxidative stress-related adverse outcome Science Tot. Env. 2023, 878: 163144. https://doi.org/10.1016/j.scitotenv.2023.163144

39. Zhou Y, He G, Jiang H, Pan K, Liu W. Nanoplastics induces oxidative stress and triggers lysosome-associated immune-defensive cell death in the earthworm *Eisenia fetida*. Env. Int. 2023, 174: 107899. https://doi.org/10.1016/j.envint.2023.107899.

Regarding the references to add to support these statements:
"In vivo uptake and transport of nanoplastics depend on their own structure and properties,
such as chemical composition" the authors can easily refer to reviews summarising these concepts as https://
doi.org/10.3390/nano11081903

The reference is already added as follows:

62. Corsi I, Bellingeri A, Eliso MC, Grassi G, Liberatori G, Murano C, Sturba L, Vannuccini ML, Bergami E. Eco-interactions of engineered nanomaterials in themarine Environment: towards an eco-design framework. Nanomaterials 2021, 11: 1903. doi.org/10.3390/nano11081903

The reference number was also updated and indicated in red.
